# The effect of modified team-based learning method on the knowledge and skills of medical emergency personnel: a clinical trial

**DOI:** 10.1097/MS9.0000000000002219

**Published:** 2024-06-04

**Authors:** Leyla Alilu, Assadollah Mohsenzadeh, Hossein Habibzadeh, Javad Rasouli

**Affiliations:** School of Nursing and Midwifery, Urmia University of Medical Sciences, Urmia, Iran

**Keywords:** clinical skills, medical emergencies, team-based learning, trauma

## Abstract

**Background:**

Trauma is one of the most important issues and problems considered in most countries in today’s modern and industrial society. Since pre-hospital care is the first component of a trauma care system, if done properly, it can reduce the problems associated with long-term disability and death due to trauma. Therefore, the present study was conducted to determine the impact of training based on a modified team-based learning (TBL) method on the skills of medical emergency personnel in managing trauma patients in 2022.

**Materials and methods:**

The present study was a two-group clinical before/after study in which 96 technicians were selected using a stratified random sampling method. The sample members were randomly divided into an intervention group and a control group. In the intervention group, skills for dealing with trauma patients were taught through a modified team-based learning method. The results were analyzed using SPSS software version 21.

**Results:**

The results of the repeated measures analysis of variance showed a significant difference between the intervention and control groups in learning skills for dealing with trauma patients (*P*<0.001), which were determined by examining the effect of test repetition and the effect of interaction. The changes in the studied variables in the TBL groups were significantly greater than those in the control group (*P*<0.001).

**Conclusion:**

The results indicate that training based on the modified team-based learning method is effective for the management of trauma patients by medical emergency personnel and improves the readiness of personnel in this field.

## Introduction

Highlights

- The modified team-based learning method training significantly improved the knowledge and skills of emergency nurses.
- The clinical trial demonstrated the modified team-based learning approach was effective among emergency nurses.
- Modified team-based learning method can improve management skills.

Medical emergency personnel play a crucial role in providing timely and effective care to individuals in critical situations^1^. Their ability to quickly assess and manage trauma cases can significantly impact patient outcomes and minimize potential complications^2^. As such, the acquisition and maintenance of adequate knowledge and clinical skills are of utmost importance in ensuring the quality of patient care and injury management^3^.

Traditionally, training programs for medical emergency personnel have relied on didactic lectures, clinical simulation training, and hands-on practice stations to impart the necessary knowledge and skills^4–6^. While these methods have proven to be valuable, there is a growing need to explore innovative and effective approaches that can enhance the learning experience and improve the retention and application of knowledge and skills^7^. One such approach that has gained recognition in medical education is the modified team-based learning (TBL) method. TBL is an active learning strategy that emphasizes collaborative problem-solving and critical thinking within small groups of learners^8^. It involves a structured process that engages learners in pre-class preparation, readiness assurance testing, and application activities. Several studies have investigated the effectiveness of TBL in various educational settings, demonstrating its positive impact on knowledge acquisition, critical thinking skills, and learner engagement^9^. However, limited research has focused specifically on the application of the modified TBL method in the context of training medical emergency personnel in trauma management.

Considering the importance of the role and performance of paramedics in trauma management and the fact that there are few studies on the effect of the TBL approach and that they were mostly conducted with students, previous studies on this method have used clinical simulation training, interactive lectures, hands-on practice stations^10^, and scenario-based training^11^, to teach trauma management, because adequate knowledge and clinical skills are critical to the quality of patient and injury care, this study aims to fill this gap by examining the effect of the modified TBL method on the knowledge and skills of medical emergency personnel in trauma management. By utilizing a team-based approach, this method is expected to promote active participation, collaboration, and problem-solving abilities among the learners. By investigating the impact of the modified TBL method on emergency medical personnel’s knowledge and skills in trauma management, this study aims to contribute valuable insights to medical education and potentially inform the development of more effective training programs for this critical workforce. The findings may have implications for improving patient care, reducing complications, and ultimately enhancing the overall quality of emergency medical services.

To ensure the quality and transparency of this study, the CONSORT 2010 checklist will be employed to guide the reporting process. This checklist provides essential items that should be included in reports of randomized controlled trials (RCTs) and offers a standardized format for documenting the flow of participants throughout the study^12^ (see Supplementary File, Supplemental Digital Content 1, http://links.lww.com/MS9/A505). Our hypotheses were:

- Participants who receive modified team-based learning method will have greater score in knowledge than participants in the control group.
- Participants who receive modified team-based learning method will have greater score in skills than participants in the control group.

## Method

### Research design

A single-blinded, parallel, randomized controlled trial (IRCT20161116030926N5) was designed to achieve the research objectives from June 2022 to July 2022.

### Participants

Ninety-six medical emergency personnel were selected for this clinical trial using a stratified random sampling method based on the population ratio of each group. The sample size was determined using a formula for comparing two averages with 99% confidence (Z_(1−α/2)=2.575 for 90% power (Z_(1−β)=1.28), and taking into account the results of a previous study by Babanazari *et al.* ^13^. The study included individuals with at least a diploma in nursing related to emergency medicine, who had not previously participated in team-based training workshops, and who were currently working in the operational area with valid work selection certificates. The exclusion criteria included unwillingness to participate in the study for any reason and missing more than two hours of the workshop.


$$n1=n2=\frac{{\left(Z_{1-\alpha /2\;}\;+\;Z_{1-\beta \;}\right)}^{2}\left({S_{1}}^{2}+{S_{2}}^{2}\right)}{{\left(\mu_{1}-\;\mu_{2}\right)}^{2}}$$

$$n1=n2=\frac{{\left(2/575\;+1/28\right)}^{2}\left({1/85}^{2}\;+\;{1/79}^{2}\right)}{{\left(12/38–10/92\right)}^{2}}\approx 48$$

### Sampling

The sampling process involved random stratification, where the list of personnel working in emergency medicine (143 participants) was divided by degree earned, and then the samples (96 participants) were randomly divided according to the population ratio of each group. The groups consisted of certified public health nurses (10 participants), emergency medicine personnel (48 participants), news service personnel (10 participants), and nursing personnel (28 participants). Participants were allocated to either the control group (C) or intervention group (G) by selecting one of two opaque sealed envelopes containing the group labels. Those who chose (C) were assigned to the control group, while those selecting (G) were placed in the intervention group. This method effectively prevented any influence or anticipation of group assignments by both researchers and participants, thereby reducing selection biases. By employing random assignment and concealment, the study’s internal validity was bolstered, promoting group comparability and reinforcing the causal link between the intervention and observed outcomes. For the intervention group, small groups of 5–6 participants were formed through simple random drawing. Recruitment started on 15 June 2022 and ended on 17 July 2022.

### Data collection tool

The data collection tool included a demographic questionnaire, a trauma exposure knowledge questionnaire, and a trauma exposure clinical skill checklist. The demographic questionnaire gathered information on age, marital status, education level, field of study, type of employment, work experience, and previous training in trauma management. The knowledge questionnaire and clinical skill checklist were designed by Shakri *et al.* ^14^ in 2013 and were validated and reliable. The knowledge questionnaire consisted of 50 multiple-choice questions, with each correct answer scored as 1 and each incorrect answer scored as 0. The scores ranged from 0 to 50 and were categorized as poor (0–16), average (17–33), and good (34–50). The reliability of the knowledge questionnaire was evaluated using the Kuder-Richardson formula 20, which yielded a score of 0.75 in the pre-test. The reliability of the Trauma Exposure Knowledge Questionnaire was evaluated using the Kodar-Richardson formula 20, which is equivalent to Cronbach’s alpha. The reported reliability score of 0.75 in the pre-test indicates a moderate level of internal consistency. This suggests that the questionnaire items are reasonably consistent in measuring trauma exposure knowledge. Based on the description, the Trauma Exposure Knowledge Questionnaire appears to have content validity. It assesses knowledge related to pre-hospital support for trauma patients and pre-hospital care of injuries. The inclusion of multiple-choice questions and scoring each correct answer (1) and incorrect answer (0) provides a standardized approach to measure the participants’ knowledge. The classification of scores into three categories (poor, average, and good) also suggests some level of construct validity, as it aligns with the expected proficiency levels based on the score ranges.

The clinical skill checklist included nine pre-hospital trauma care skills, each scored as either 1 (performed) or 0 (not performed). The scores were classified as poor (0–45), average (46–92), and good (93–139). The reliability coefficient for all skills in this checklist was calculated to be 0.80^15^.

### Intervention

To prevent staff work shifts from coinciding with training sessions, the dates of the training sessions coincided with participants’ shift schedules. Once established, participants of the intervention group were randomly assigned to small groups of 5–6 participants by lottery. In the intervention group, trauma patient management training using the TBL method was conducted in three stages. The first stage consisted of preparation for the class. At the end of the introductory session, the details of the teaching method and the training objectives were explained, and the desired resources in accordance with the content of the training for each of the teaching sessions were presented to the staff. The training materials in the form of brochures, slides, and videos were provided. The modified team-based learning method in this study was specifically tailored to accommodate the Iranian cultural context. Several modifications were implemented to align the approach with the cultural norms and preferences of the participants. Firstly, the team formation process took into consideration the cultural values of collectivism and group harmony by ensuring a balanced mix of students from different backgrounds and abilities within each team. Additionally, the content and examples used during the learning activities were carefully selected to reflect the Iranian cultural context, incorporating local references and scenarios that resonated with the students’ experiences. Moreover, the assessment methods were adjusted to include culturally relevant evaluation criteria, allowing for a more comprehensive and accurate assessment of the students’ learning outcomes. Overall, these modifications aimed to create a culturally sensitive and inclusive learning environment, fostering active engagement, collaboration, and effective knowledge acquisition among the participants.

Cultural adaptations to the TBL approach involve modifying the methodology to suit the cultural norms, values, and preferences of the specific population. This includes adjusting language, content, group dynamics, learning styles, respecting diversity, assessment methods, and educator training to ensure the approach aligns with the cultural context, enhancing the learning experience for participants from diverse backgrounds.

The first 20–30 min of the second phase of each training session were devoted to assessing the readiness of the participants. At the beginning of the training session, the intervention group was presented with an individual readiness assessment test that included 10–20 questions with 4 options related to the training content. This test took 10–15 min to complete. Then, each group of 5–6 people chose a leader, and for the next 10–15 min, the test to ensure group readiness was administered by answering the same questions as a group. After the questions were answered, the instructor reviewed their answers and clarified any concepts that the participants could not answer. The third and most important phase involved hands-on training in dealing with trauma patients. Each group was given an assignment by presenting a case related to the basic concepts and skills implementation of the training topic. All groups worked on their task for 5–15 min. Groups were able to bring the necessary resources, such as laptops, to class. Each team was identified by a flag that the teams raised when they reached their answer. When the flags were raised, the teacher asked the teams to present their answer. When the time was up, the teacher asked the teams to demonstrate their skills. The answers were presented in a variety of hands-on ways, such as on a patient screen, mannequin, classroom whiteboard, etc., and the groups discussed and provided feedback to each other. Finally, the teacher reviewed each team’s homework, collected their best answers, and implemented them. The last 30 min of the third stage were spent on practical training and addressing personnel issues. Participants in the control group only received routine care. One week and one month after the completion of the educational intervention, the post-test for measuring the knowledge and skills of dealing with trauma was held again in the two intervention and control groups. The author, who has 18 years of training experience, was an educator.

Statistical analysis was performed using SPSS 21 software. The normality of the data was checked using the Kolmogorov–Smirnov test, and parametric tests were used for analysis since the data followed a normal distribution (*P* > 0.05). Repeated measure ANOVA was employed to compare the averages at the three time points: before the intervention, 1 week after, and 1 month after (*P*=0.05). All analyses were performed by a researcher who was blind to the data.

## Results

**Figure 1:**
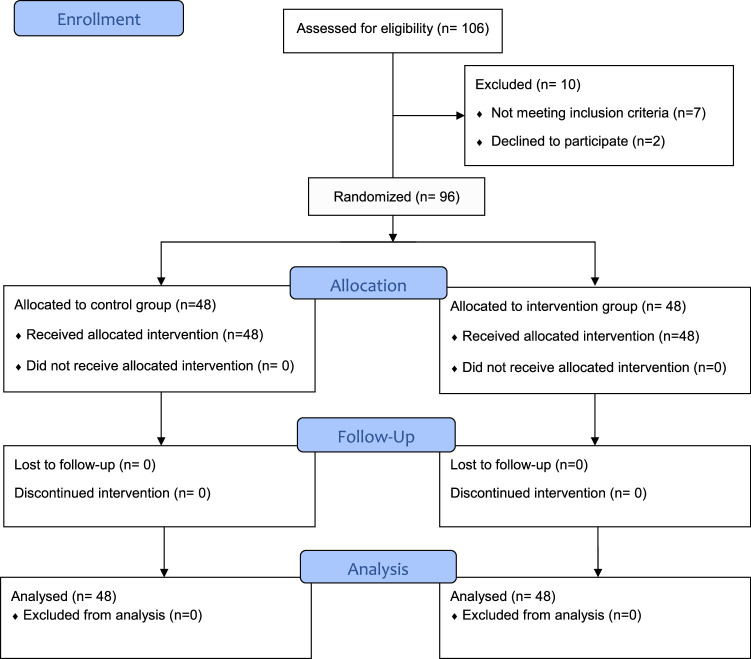
CONSORT flow diagram of study.

In this study, the average age of participating medical emergency personnel was 28.96±2.36 years. Additionally, the average work experience among participating medical emergency personnel was 5.04±2.23 years (Figure 1). Among the participants, 50% (48 people) had a field of study in emergency medical technician, 10.4% (10 people) were anesthesiologists, 29.2% (28 people) were nurses, and 10.4% (10 people) were paramedics (Table 1). The results also indicated that there was no statistically significant difference between the two groups in terms of demographic information (*P* >0.05). The mean score of trauma exposure knowledge increased in the intervention group after the educational intervention. However, in the control group, there was no difference between the average score of the pre-test, the post-test, and the 1-month follow-up (Table 2).

**Table 1:** Comparison of the demographic characteristics of the research units between the intervention and control groups.

	Intervention		Control		
Variables	Number	Percent	Number	Percent	Results
Material status					
Single	28	53.3	31	64.6	*P*=0.529
Married	20	41.7	17	35.4	
Education					
Secondary	5	10.4	5	10.4	*P*=0.910
Bachelor	18	37.5	20	41.7	
Master	25	52.1	23	47.9	
Major					
Emergency medicine	24	50	24	50	*P*=1.000
Anesthesia nurse	5	10.4	5	10.4	
Nurse	14	29.2	14	29.2	
Unlicensed assistive personnel	5	10.4	5	10.4	
Passing the trauma training course					
Yes	8	16.7	7	14.6	*P*=0.779
No	40	83.3	41	85.4	

**Table 2:** The average score of trauma exposure knowledge in the two intervention and control groups

Knowledge	Average and standard deviation of knowledge score in 3 time periods		
Group	Before the intervention	After the intervention	One month after the intervention
Intervention	30.16±3.03	40.22±2.69	39.14±1.86
Control	31±3.17	31.50±2.89	31.20±2.47

According to Table 2, the average knowledge score before the intervention, 1 week after the intervention, and 1 month after the intervention in the intervention group were (30.16±3.03), (40.22±2.69), and (39.14±1.86), respectively. In the control group, the average scores were (31.0±3.17), (31.50±2.89), and (31.20±2.47).

Repeated measures ANOVA was used to examine the knowledge score at 3 time points between the control and intervention groups, and the results are shown in Table 3.

**Table 3:** Repeated measurement analysis of knowledge score in 3 time points between intervention and control groups.

Knowledge	Sum of squared error	F	*P*	Partial Eta squared
Main effect (time)	1582.94	270.327	<0.001	0.742
Main effect (intervention)	668.519	122.210	<0.001	0.565
The interaction of time with the intervention	1351.965	230.882	<0.001	0.711

Due to the nature of the dependent variable, measurements were repeated at earlier, 1-week, and 1-month time points. Repeated measures analysis was used for comparisons between the two groups. According to the required assumptions, the corresponding analysis shows that the assumption of sphericity between values does not hold (*P*<0.001). Geisser’s correction was used. The results show that the effect of time is significant (Eta2=0.742, *P*<0.001). This means that changes in participants’ knowledge scores increase over time, and these changes are statistically significant. The interaction effect of time and group (intervention) is also significant (Eta2=0.711, *P*<0.001). This means that the changes in knowledge scores over time show significant differences depending on the level of the groups studied (intervention and control). Due to the presence of a significant interaction between the group variable and time, reanalysis was performed separately according to the levels of the group variables (control and intervention), and the results showed that the assumption of standard sphericity was not met for the control group. Therefore, the Greenhouse–Geisser test was used, which showed that the trend of changes over time was not significant. For the intervention group, the assumption of sphericity was not met (*P*=0.003, χ2=11.644), so the Greenhouse–Geisser test was used, which showed that the changes had a significant increasing trend over time (Eta2=0.898, *P*<0.001), indicating the effectiveness of the intervention applied.

The results showed that in the intervention group, the average knowledge scores before the intervention, 1 week later, and 1 month later were statistically significant (*P*<0.05). This means that the knowledge score increased significantly one week after the intervention. In the control group, there was also no statistically significant difference between the average knowledge scores before the intervention and 1 week and 1 month later (*P* > 0.05).

Table 4 shows that the mean score of total competence before the intervention, one week after the intervention, and 1 month after the intervention were (72.52±5.86), (89.86±3.86), and (111.66±4.36) in the intervention group, and (70.97±5.34), (71.20±5.70), and (70.79±5.70) in the control group. Repeated measures analysis of variance was used to examine the total skill scores at three time points between the control and intervention groups, and the results are shown in Table 5. The results are presented in Table 5. Additionally, Bonferroni multiple comparisons were used to compare the average scores of the total skills within the control and intervention groups, and the results are shown in the following table.

**Table 4:** The average score of total skill, between two intervention and control groups, before, 1 week and 1 month after the implementation of the training.

Total skill	Average and standard deviation of total skill score in 3 time periods		
Group	Before the intervention	After the intervention	One month after the intervention
Intervention	72.52±5.86	114.89±3.86	111.66±4.36
Control	70.5±7.34	71.20±5.70	70.79±5.70

**Table 5:** Repeated measurement analysis of total skill score in 3 time points between intervention and control groups.

Total skill	Sum of squared error	F	*P*	Partial Eta squared
Main effect (time)	26 769.271	2233.963	<0.001	0.960
Main effect (intervention)	19 770.473	859.911	<0.001	0.901
The interaction of time with the intervention	26 650.340	2224.038	<0.001	0.959

From Table 5, it can be seen that in the intervention group, the mean scores for total competence before the intervention, 1 week later, and 1 month later were statistically significant. This indicates that the total skill score increased significantly one week after the intervention. In the control group, the average scores of total skills before the intervention, 1 week after the intervention, and 1 month after the intervention were not significantly different (*P*<0.05).

## Discussion

The current study aimed to investigate the effect of a modified team-based learning method on the knowledge and skills of medical emergency personnel in dealing with trauma patients.

The results of this study indicated that the participants had subpar performance in terms of knowledge and skills related to trauma patient management before the educational intervention, achieving an average score. This finding is consistent with a study conducted in Sweden that evaluated pre-hospital emergency workers’ assessment of trauma patients, which highlighted the technicians’ difficulties and the need for more practical skills and training^16^. Similarly, Kumar *et al.* ^17^ found that the average performance score of healthcare providers in pre-hospital and emergency care was below average, emphasizing the need for improvement. Another study by Norouzinia *et al.* ^18^ evaluated the knowledge and clinical skills of emergency medicine students in trauma management, revealing skill gaps in areas such as limiting movement of injured long bones and trauma patient examination. Considering the critical role of pre-hospital emergency technicians as first responders and the high incidence of accidents, continuous training and retraining programs in trauma patient management are expected to enhance their ability to provide optimal and standardized care, ultimately reducing the rates of death and disability among the injured. Dadashzadeh *et al.* ^19^ found that neck collar and backboard usage was limited among Tabriz pre-hospital emergency workers, highlighting the importance of further investigation and trauma patient management training programs for employees.

Furthermore, the results of the present study showed no statistically significant difference in the average scores of knowledge and skills related to trauma patients between the two groups before the intervention. However, after the intervention, a statistically significant difference was observed. Specifically, the average scores of knowledge and skills in the group that received the modified team-based educational intervention increased compared to the control group, indicating the effectiveness of this training method for emergency medical personnel. Similar findings have been reported in previous studies. For instance, Zoghib investigated the satisfaction and performance of second-year medical students in a pharmacology unit taught using a modified team-based method, and the results showed higher group evaluation scores compared to individual evaluation and higher scores in TBL compared to lecture-based learning^20^. Another study by Zoghib *et al.* ^21^ implemented TBL in rational drug prescribing sessions for fourth-year medical students, leading to high student satisfaction and improved performance. Weiner *et al.* ^22^ examined the effect of TBL on teaching key subjects to first-year medical students at the University of Vienna and found that students responded positively to this learning method, which enhanced their success in challenging tests.

Although our study did not directly compare the modified team-based method with other teaching approaches such as lectures or scenario-based teaching, similar to other studies using team-based methods, we observed a significant improvement in grades after the educational intervention. These findings suggest that learners’ performance in TBL courses consistently improves. This is in line with Zaghib *et al.* ^20^‘s study, which concluded that team-based learning enhances students’ performance in pharmacology education. Coles and colleagues also emphasized the positive impact of team learning on the academic performance of medical students^23^. However, it should be noted that some studies have reported no significant increase in students’ scores. Margolis *et al.* ^24^ stated that more research is needed to confirm the effectiveness of team-based training in improving learners’ performance. Norouzinia *et al.* ^18^ attributed the lack of improvements to specific issues in their study rather than dismissing the overall effectiveness of the approach.

One potential explanation for the high post-test scores in our study could be the retention of content facilitated by TBL. As the post-test was administered 1 week and 1 month after the intervention, and the participants were unaware of it, they may have retained the knowledge better. This finding aligns with other studies demonstrating that learning is better and longer retained after TBL^20,22,25^.

Acknowledging several limitations in our study is crucial for a comprehensive understanding of the research outcomes. Firstly, the absence of blinding participants to the intervention introduces the potential for performance bias. Participants aware of receiving the team-based learning intervention might exhibit heightened motivation or engagement, potentially overestimating the intervention’s effectiveness and compromising the study’s internal validity.

Another limitation pertains to selection bias. Non-random selection could lead to systematic differences between groups, affecting the generalizability of study findings. Self-selection of participants with specific characteristics or motivations might create a biased sample that does not accurately represent the broader population.

Additionally, while a small number of TBL sessions enhanced participants’ learning, increasing the session count could yield more substantial results. The strength of the study lies in the observed improvements in learning and material retention 1-month post-intervention, reflecting significant enhancements in trauma patient management scores and the sustainability of course material.

Furthermore, participants’ shift schedules interfering with training sessions and technicians’ concurrent work at the base, aligning training class dates with participants’ shifts, posed a challenge. The lack of gender diversity among participants limits the findings’ generalizability, as the results may not reflect the broader population. It’s crucial to recognize that the study’s outcomes may only be applicable to the specific male participant group studied, cautioning against broad extrapolation to populations with diverse gender compositions.

## Conclusion

According to the findings of the research, it can be said that with the modified team-based training method, the knowledge and skills of the personnel in dealing with trauma patients can be improved, and the medical emergency personnel can be improved by increasing and strengthening the scientific and practical level. They can greatly reduce the disadvantages of dealing with trauma patients and thereby improve their clinical outcomes because the correct action will play a very important role in the treatment and final rescue of patients and will reduce irreparable complications in the family and society.

## Ethical approval

Ethical approval for this study (Ethical Committee IR.UMSU.REC.1398.434) was provided by the Ethical Committee of Urmia University of Medical Sciences, Iran on 10 November 2021.

## Consent

Written informed consent was obtained from the patient for publication of this study. A copy of the written consent is available for review by the Editor-in-Chief of this journal on request.

## Source of funding

Not applicable.

## Author contribution

L.A. and A.M.: study concept, data collection, writing the paper and making the revision of the manuscript following the reviewer’s instructions. L.A. and H.H.: study concept, reviewing and validating the manuscript’s credibility.

## Conflicts of interest disclosure

The authors declare that they have no conflicts of interest.

## Research registration unique identifying number (UIN)

1. Name of the registry: Iranian Registry of Clinical Trials
2. Unique identifying number or registration ID: IRCT20161116030926N5.
3. Hyperlink to your specific registration (must be publicly accessible and will be checked): https://irct.behdasht.gov.ir/search/result?query=IRCT20161116030926N5.

## Guarantor

Leyla Alilu.

## Data availability statement

The datasets generated during and/or analyzed during the current study are available upon reasonable request.

## Provenance and peer review

Not commissioned, externally peer-reviewed.

## Supplementary Material
